# Supplementary material for: Bridging the microstructural gap in human connectomics using hierarchical phase-contrast tomography as a reference for diffusion MRI in the human brain
Source: bioRxiv. 2026 Apr 6:2026.04.02.715729. Preprint. [Version 1] doi: 10.64898/2026.04.02.715729 (PMC13081970; doi:10.64898/2026.04.02.715729)
Supplement: Supplement 1 [file NIHPP2026.04.02.715729v1-supplement-1.pdf]

bioRxiv preprint doi: https://doi.org/10.64898/2026.04.02.715729; this version posted April 6, 2026. The copyright holder for this preprint (which was not certified by peer review) is the author/funder, who has granted bioRxiv a license to display the preprint in perpetuity. It is made available under aCC-BY 4.0 International license.

Table A1 HiP-CT scanning parameters.

Sample ID	Voxel (μm)	Prop. dist. (m)	Attenuator	Energy (keV)	Lat. FOV (mm)	Proj.	Expo (ms)	Accum.	Acq. mode	Scan time (s)	N scans	Vert. FOV (mm)	Vert. step (mm)
I58	15.13	15	Sapphire 10mm, SiO_2 15mm block	90	142.42	15000 x 2	14	5	quarter	1071	32	6.05	5
LADAF-2021-17	6.54	10	Mo 0.21mm	120	25.15	6000	60	4	half	369	9	13.4	7

Appendix A Supplementary Information

A.1 HiP-CT Acquisition Parameters

Table A1 details the specific synchrotron X-ray phase-contrast imaging setups used for the hierarchical scanning of the brain samples. Because HiP-CT relies on a propagation-based phase-contrast setup , parameters were specifically optimized for each target resolution. Data for the macroscopic overview scan (Sample I58) was acquired at a 15.13 µm voxel size using an energy of 90 keV, requiring 32 vertical scans to capture the extensive 142.42 mm lateral field of view. In contrast, the high-resolution, localized volume (Sample LADAF-2021-17) was acquired at a 6.54 µm voxel size at 120 keV, utilizing a shorter propagation distance (10 m) and a tighter 25.15 mm field of view. Both acquisitions utilized multiple vertical steps and optimized attenuators to manage beam flux and minimize artifacts across the sample volumes.

A.2 Human-in-the-loop Vessel Segmentation

As noted in the main text, HiP-CT is a non-targeted imaging modality that naturally resolves a variety of structural features alongside white matter, most prominentl y dense vascular networks spanning from large vessels down to micro vasculature [68, 69]. Because these vascular structures exhibit strong tubular or sheet-like geometries, they generate pronounced spatial gradients in the phase-contrast images [60]. During structure tensor analysis, these non-axonal gradients can artificially inflate local eigenvalues, thereby introducing spurious anisotropy and biasing the orientation measurements intended to characterize white matter architecture.

To quantitatively assess and control for this confounding factor, we developed a pipeline to segment and mask the vasculature prior to tractography. This section details the human-in-the-loop (HITL) deep learning framework utilized to isolate these networks. By combining the high-throughput inference capabilities of a Convolutional Neural Network (CNN) with iterative human expert review, we achieved accurate, multi-scale vascular segmentation while minimizing the prohibitive manual annotation typically required for massive tomographic datasets.

bioRxiv preprint doi: https://doi.org/10.64898/2026.04.02.715729; this version posted April 6, 2026. The copyright holder for this preprint (which was not certified by peer review) is the author/funder, who has granted bioRxiv a license to display the preprint in perpetuity. It is made available under aCC-BY 4.0 International license.

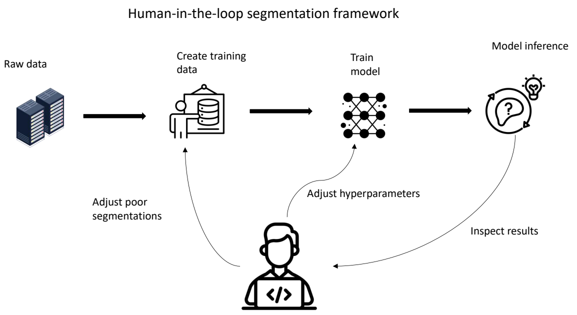

Fig. A1 Human-in-the-loop (HITL) workflow for multi-scale vascular segmentation in HiP-CT data. The iterative process leverages a 2.5D U-Net within the ORS Dragonfly Segmentation Wizard. Initial manual annotations of a 6.54 µm VOI from the pons establish a baseline model. Subsequent cycles of full-volume inference, human expert review, targeted error correction, and training set expansion progressively refine the model's ability to isolate complex, multi-scale vascular networks from the surrounding structural anatomy.

**Model Architecture**	**Training Parameters**	**Data Augmentation**
Depth level: 6	Batch size: 512	Horizontal/vertical flips
Initial filter count: 64	Patch size: [64, 64, 1]	Rotation: 180.0°
Input dimension: 2.5D, 3 slices	Stride ratio: 0.25<br>Loss function: Binary cross-entropy	Zoom: [0.9, 1.1]
	Optimization: Adadelta<br>Epochs: 100	Shear: 2.0

Table A2 Convolutional Neural Network architecture and training hyperparameters. Parameters define the 2.5D U-Net deployed for the semantic segmentation of vasculature. The architecture utilizes a depth of 6 and 3-slice input dimensions to capture continuous 3D spatial context, while extensive data augmentation (rotations, flips, zooming, and shearing) was applied to force model generalization across the diverse morphological scales of the vascular network.

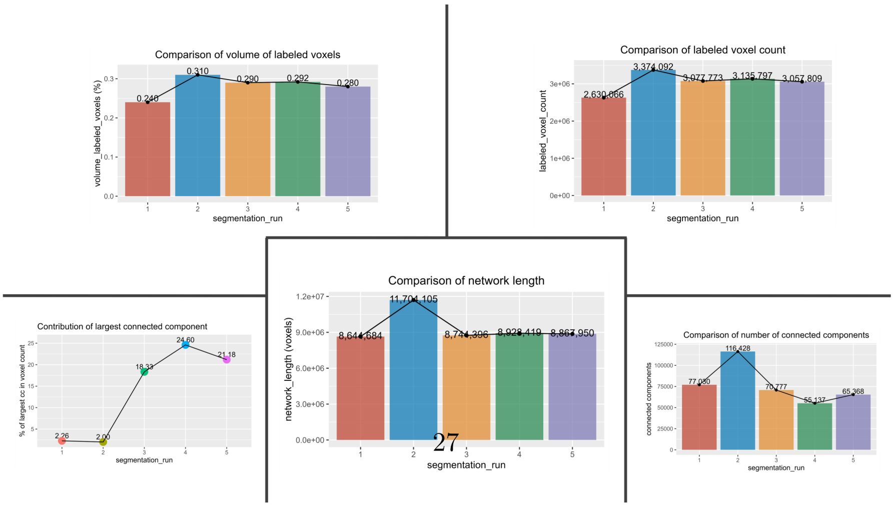

Fig. A2 Evolution of quantitative vascular segmentation metrics across iterative training rounds. The graph tracks key performance indicators—including validation Dice score, connected component count, and total labeled voxel volume—as the human-annotated training set expanded from 7 to 53 slices. Performance metrics peaked at the fourth training iteration (33 slices) before declining at the fifth iteration, indicating the onset of diminishing returns and model overfitting.

bioRxiv preprint doi: https://doi.org/10.64898/2026.04.02.715729; this version posted April 6, 2026. The copyright holder for this preprint (which was not certified by peer review) is the author/funder, who has granted bioRxiv a license to display the preprint in perpetuity. It is made available under aCC-BY 4.0 International license.

Training Round	Epochs Trained	Validation Dice
1	100	0.8325
2	100	0.7986
3	100	0.8778
4	100	0.9264
5	100	0.9186

Table A3 Summary of validation performance per HITL training iteration. Validation Dice scores were recorded after 100 epochs for each of the five training rounds. The model from Round 4 achieved the highest quantitative accuracy (Dice = 0.9264) and was subsequently selected to generate the final vascular masks for the structure tensor analysis pipeline.

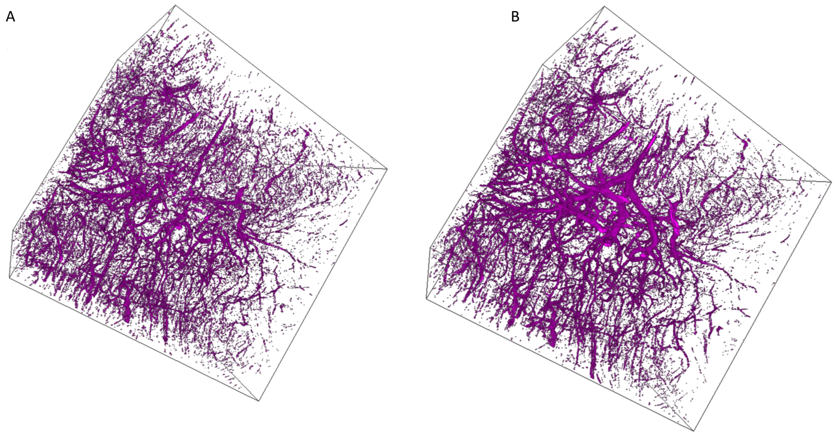

Fig. A3 Qualitative improvement in multi-scale vascular network segmentation. (a) The segmented vessel network following the initial training iteration captures prominent large vessels but suffers from severe structural fragmentation and fails to resolve microvasculature detail. **(b)** The optimized network from the fourth training iteration demonstrates significantly improved anatomical plausibility. It successfully resolves both massive tubular vessels and the dense, continuous microvascular bed.

A.3 Masking out vasculature effects

As detailed in the main text, safely removing the confounding influence of vasculature from the structure tensor analysis requires careful timing within the computational pipeline. Because the structure tensor calculates orientation based on spatial intensity derivatives, simply zeroing out the vessels in the raw image volume (Pre-Structure Tensor Masking) creates sharp, artificial boundaries between the intact tissue and

bioRxiv preprint doi: https://doi.org/10.64898/2026.04.02.715729; this version posted April 6, 2026. The copyright holder for this preprint (which was not certified by peer review) is the author/funder, who has granted bioRxiv a license to display the preprint in perpetuity. It is made available under aCC-BY 4.0 International license.

the empty vessel voids. The derivative of this artificial step-edge manifests as a massive, spurious gradient. To visualize why this approach is detrimental, this section demonstrates the artificial edge effects caused by pre-masking compared to the clean signal suppression achieved by applying the mask to the spatial derivatives directly (Gradient-Level Masking).

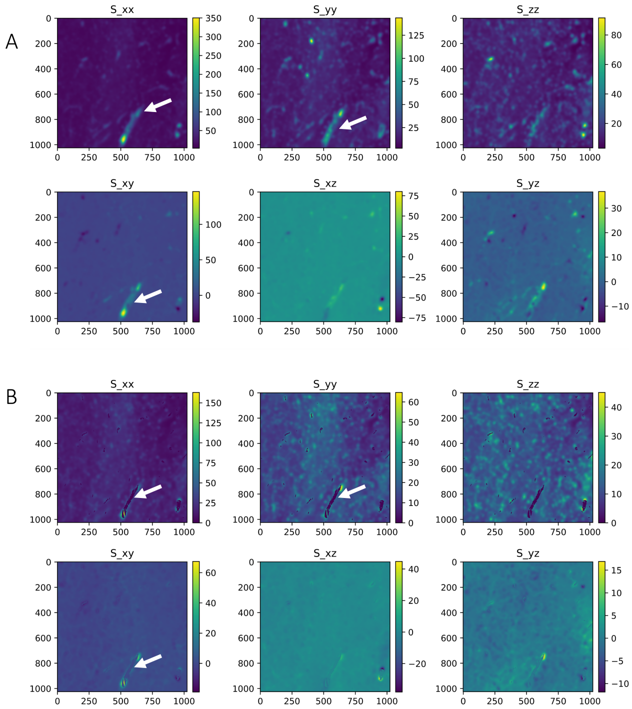

Fig. A4 Comparison of vasculature masking strategies on structure tensor gradient computation. **(A)** Pre-Structure Tensor Masking: Removing vessels directly from the raw Volume of Interest (VOI) creates sharp artificial boundaries, inadvertently generating massive, spurious gradients (high intensity) around the vessel voids. **(B)** Gradient-Level Masking: Applying the mask after computing the spatial derivatives effectively suppresses the vascular signal, resulting in the intended low-gradient regions without introducing boundary artifacts.

A.4 Effects of vasculature on white matter fiber anisotropy

We extended vasculature effect analysis to quantify the specific effects of vasculature on white matter anisotropy and shape metrics, namely Fractional Anisotropy (FA), linearity (fiber-like symmetry), and planarity (plane-like symmetry). Voxel-wise comparisons of these metrics, computed both with and without gradient-level vascular masking, are presented as violin plots in **Figure A5 a, b, c**.

bioRxiv preprint doi: https://doi.org/10.64898/2026.04.02.715729; this version posted April 6, 2026. The copyright holder for this preprint (which was not certified by peer review) is the author/funder, who has granted bioRxiv a license to display the preprint in perpetuity. It is made available under aCC-BY 4.0 International license.

The violin plots reveal highly similar and largely symmetric distributions across both conditions, exhibiting comparable interquartile ranges for FA, linearity, and planarity. This visual similarity suggests that the overall population characteristics of these shape metrics within white matter voxels remain largely unperturbed by the presence of unmasked vessels. While statistical testing (Wilcoxon signed-rank and paired t-tests) yielded statistically significant differences (p ¡ 0.05), this is primarily an artifact of the massive sample size inherent to high-resolution, voxel-wise tomographic data. Crucially, the effect sizes for all morphological metrics were consistently trivial ($Cohen'sd < 0.2$). Therefore, while the influence of vasculature on local anisotropy and shape measures is statistically detectable, its actual magnitude and practical impact on the underlying white matter characterization are negligible.

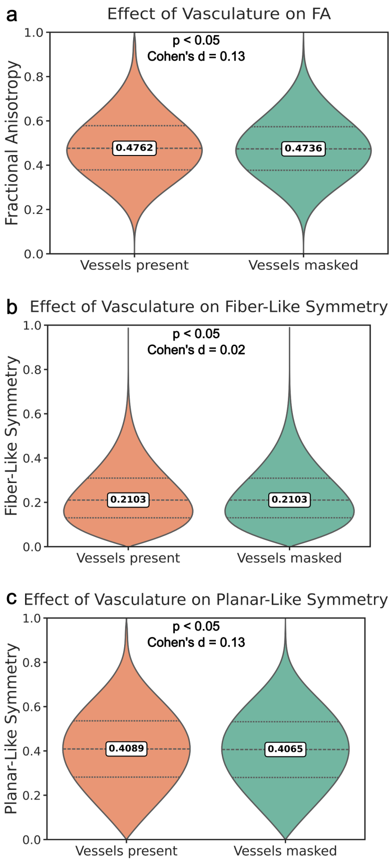

Fig. A5 Impact of vascular masking on white matter anisotropy and shape metrics. Violin plots display the voxel-wise distributions of **(a)** Fractional Anisotropy (FA), **(b)** linearity, and **(c)** planarity computed with and without gradient-level vascular masking. While massive voxel counts drive statistically significant differences (p ¡ 0.05), the highly overlapping distributions and trivial effect sizes (Cohen's d ¡ 0.2) demonstrate that vasculature has a negligible magnitude of effect on the overall characterization of white matter morphology.

bioRxiv preprint doi: https://doi.org/10.64898/2026.04.02.715729; this version posted April 6, 2026. The copyright holder for this preprint (which was not certified by peer review) is the author/funder, who has granted bioRxiv a license to display the preprint in perpetuity. It is made available under aCC-BY 4.0 International license.

A.5 Effects of artifacts on HiP-CT STA based tractography

The use of Fomblin, a perfluoropolyether fluid routinely employed during ex vivo MRI to prevent tissue dehydration and minimize magnetic susceptibility artifacts, introduces specific challenges for correlative HiP-CT imaging. Despite extensive sample preparartion and washing protocols following the dMRI scans, residual fomblin can remain stubbornly trapped within deep sulci and vascular cavities **Figure A6 a, b**. Because fomblin has a vastly different X-ray attenuation coefficient profile and electron density compared to the surrounding fixed brain tissue, these residual droplets create sharp, high-contrast structural boundaries. During tomographic reconstruction, these sharp density transitions generate severe streak artifacts that propagate linearly across the adjacent tissue **Figure A6 c, e**. Because structure tensor analysis relies fundamentally on local spatial intensity gradients to estimate fiber orientation, these streak artifacts act as a massive confounding factor and are misinterpreted as highly coherent biological tissue. Consequently, this leads to the generation of dense, spurious streamlines that align with the artificial gradient trajectories **Figure A6 d, f**. These observations reinforce the necessity of careful HiP-CT sample preparation, reconstruction and quality control.

A.6 Beyond dMRI equivalent resolution

To demonstrate the scalable potential of the pipeline beyond matched dMRI resolution, we repeated the structure tensor analysis using 400 µm supervoxels instead of the 800 µm supervoxels used in the main text. The high resolution 15 µm field was first grouped into 400 µm supervoxels, after which spherical histograms were constructed and fitted with 8th-order spherical harmonics to obtain fODFs, exactly as described for the 800 µm case. All other tractography parameters remained identical. The resulting 400 µm tractogram are shown in Supplementary Figure A7. Reducing the supervoxel size preserved the overall bundle organization and global topological agreement with the 800 µm results while visually yielding denser, continuous and spatially confined streamlines.

bioRxiv preprint doi: https://doi.org/10.64898/2026.04.02.715729; this version posted April 6, 2026. The copyright holder for this preprint (which was not certified by peer review) is the author/funder, who has granted bioRxiv a license to display the preprint in perpetuity. It is made available under aCC-BY 4.0 International license.

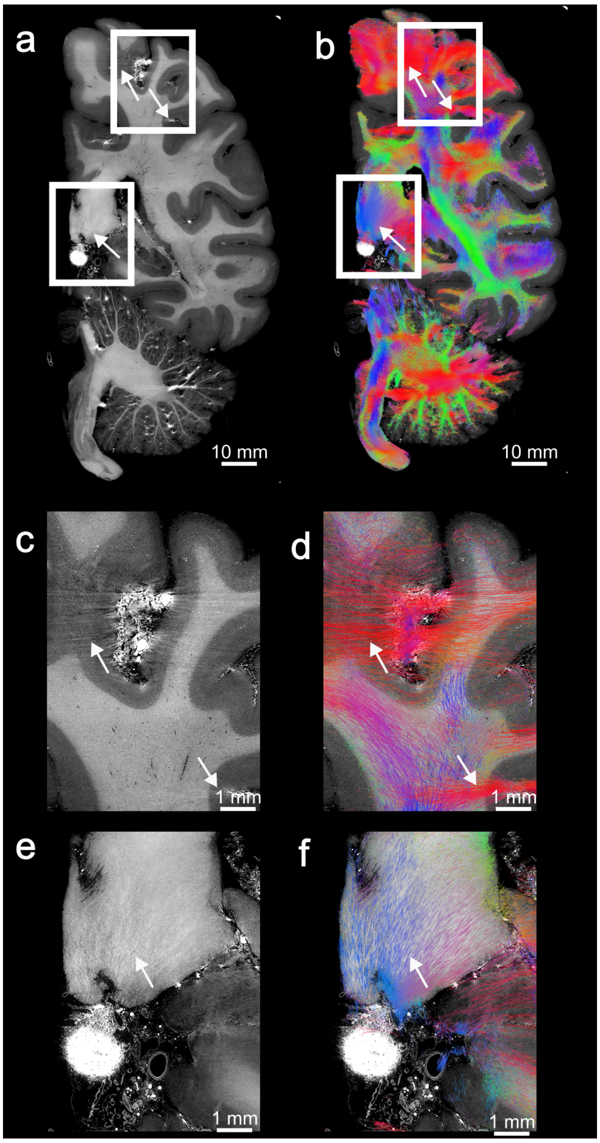

Fig. A6 Impact of residual Fomblin and tomographic streak artifacts on HiP-CT structure tensor tractography. (a, b) Macroscopic coronal cross-sections of HiP-CT intensity (a) and corresponding structure tensor tractography (b). Highly attenuating Fomblin fluid, retained from prior ex vivo dMRI sample preparation, pools in macroscopic cavities and deep sulci. **(c, e)** Magnified regions revealing severe, high-frequency streak artifacts propagating from the dense fomblin deposits during tomographic reconstruction. **(d, f)** The corresponding structure tensor tractography for these magnified regions. Because the algorithm relies on local spatial derivatives, it mathematically misinterprets the strong, artificial intensity gradients of the streak artifacts as highly coherent biological tissue. This results in the generation of dense, spurious streamlines (white arrows) that align with the artifactual streaks, most notably the dense horizontal tracts in (d), locally obscuring and overwriting the true underlying white matter architecture.

bioRxiv preprint doi: https://doi.org/10.64898/2026.04.02.715729; this version posted April 6, 2026. The copyright holder for this preprint (which was not certified by peer review) is the author/funder, who has granted bioRxiv a license to display the preprint in perpetuity. It is made available under aCC-BY 4.0 International license.

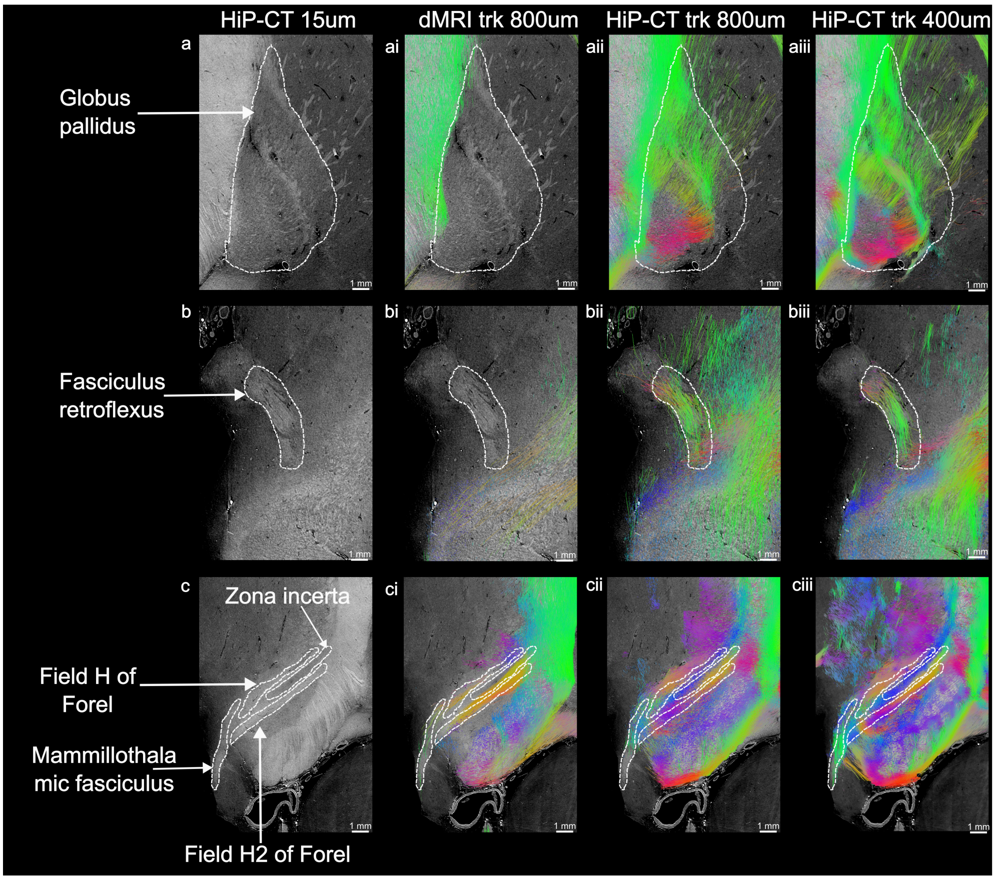

Fig. A7 Comparison of the same deep-brain regions shown in Figure 4, now including HiP-CT-STA tractography performed with 400 µm **supervoxels (rightmost column).** Reducing the aggregation scale preserved the global topological organization of the major white matter bundles while minimizing partial volume effects yielding yielding denser, continuous and spatially confined streamlines. Streamlines are colored by local orientation (Red: left–right, Green: anterior–posterior, Blue: superior–inferior).
